# Interaction of Alkannin with CPEB4 Contributes to Its Antitumor Effects in Melanoma

**DOI:** 10.3390/biom16071064

**Published:** 2026-07-21

**Authors:** Parwen Parhat, Min Li, Wenying Li, Jinyan Li, Mubarak Obulkasim, Yinglan Ma

**Affiliations:** 1 The Xinjiang Key Laboratory of Natural Medicine Active Components and Drug Release Technology, College of Pharmacy, Xinjiang Medical University, Urumqi 830011, China; parewenpalihati@stu.xjmu.edu.cn (P.P.); wenyingli599@163.com (W.L.); jinyanli1015@stu.xjmu.edu.cn (J.L.); mbrak555@163.com (M.O.); myl0713@stu.xjmu.edu.cn (Y.M.); 2 Xinjiang Key Laboratory of Biopharmaceuticals and Medical Devices, Xinjiang Medical University, Urumqi 830011, China

**Keywords:** alkannin, melanoma, CPEB4, target identification, antitumor mechanism

## Abstract

Melanoma is a highly aggressive malignancy characterized by strong invasive and metastatic potential. CPEB4 has been implicated in melanoma progression and may serve as a potential therapeutic target. Alkannin has previously been reported to exert antitumor activity against melanoma; however, its in vivo efficacy and direct molecular interaction with CPEB4 remain unclear. In this study, a subcutaneous xenograft model using BALB/c nude mice was used to assess the in vivo antitumor effects of alkannin, and CPEB4 expression was analyzed via Western blotting. DARTS, CETSA, and SPR investigations were used to elucidate the interaction between alkannin and CPEB4. In addition, stable CPEB4-knockdown A375 melanoma cells were established to examine the effects of alkannin on cell proliferation, apoptosis, cell cycle progression, migration, invasion, and downstream signaling molecules. Alkannin markedly suppressed tumor growth in the xenograft model and reduced CPEB4 expression in a dose-dependent manner compared with the model group. DARTS and CETSA demonstrated alkannin-induced stabilization of CPEB4, while SPR analysis using purified recombinant CPEB4 showed a direct physical interaction with alkannin, with micromolar affinity. At the molecular level, alkannin downregulated CPEB4 and PRC1 expression (*p* < 0.05), whereas CPEB4 knockdown markedly suppressed MITF and PRC1 (*p* < 0.05). Notably, alkannin treatment alone did not significantly alter MITF protein expression under the present experimental conditions. Alkannin exerts antitumor activity against melanoma, while its interaction with CPEB4 and the associated molecular changes may contribute to cellular responses involving proliferation, survival, migration, invasion-related phenotypes, and mitotic regulation.

## 1. Introduction

Melanoma is a highly malignant tumor originating from melanocytes and can occur in the skin, mucous membranes, and ocular tissues, with cutaneous melanoma representing the predominant subtype [1,2,3,4]. Although melanoma accounts for a relatively small proportion of all skin cancers, its marked invasiveness, high metastatic potential, and poor prognosis in advanced or metastatic disease make it a major cause of skin cancer mortality [5,6,7]. The global burden of melanoma remains substantial. According to IARC GLOBOCAN 2022, there were approximately 330,000 new melanoma cases and 58,000 melanoma-related fatalities globally in 2022 [7,8]. Melanoma pathogenesis is strongly associated with multiple risk factors, particularly ultraviolet (UV) exposure, skin phenotype, and nevus burden [9,10]. Abnormalities in driver genes such as BRAF, NRAS, and NF1, together with the consequent dysregulation of signaling pathways, including MAPK and PI3K/AKT/mTOR, are key events driving melanoma initiation, progression, and therapeutic resistance [11,12,13]. Surgical excision remains the standard treatment for early-stage melanoma [14,15]. Immune checkpoint inhibitors and BRAF/MEK-targeted combination therapy have significantly enhanced clinical outcomes for patients with unresectable or metastatic melanoma, particularly those with BRAF-mutant tumors [16,17]. However, these therapeutic strategies are still limited by adverse reactions, variable responses, limited durability of efficacy, and acquired resistance [18]. Recent investigations have demonstrated that Arnebia euchroma and its active ingredients can regulate cancer-associated malignant phenotypes through multitarget, multipathway mechanisms [19]. These compounds have demonstrated promising antitumor activity in preclinical studies and may have potential for further development as therapeutic agents for melanoma.

**Figure 1:**
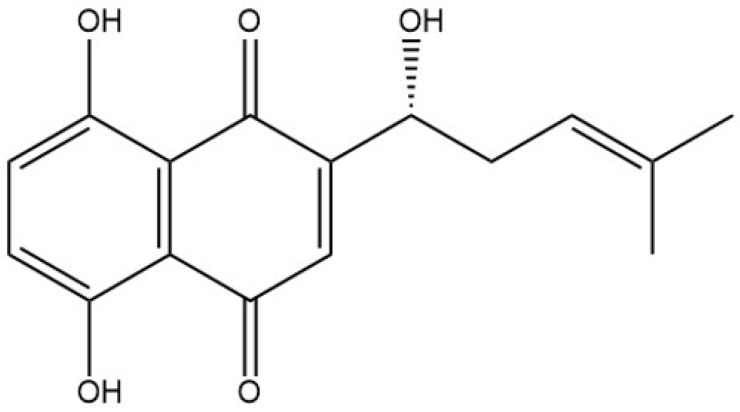
Chemical structure of alkannin (C_16_H_16_O_5_, MW:288.3).

Alkannin (Figure 1) is a natural naphthoquinone compound extracted from *Arnebia euchroma (Royle) Johnst.* and is one of its primary active components [20]. Arnebia euchroma and its derivatives possess pharmacological activities, including anti-inflammatory, antibacterial, antitumor, and wound-healing properties [21,22]. Studies have shown that acetylalkannin extracted from Xinjiang Arnebia euchroma suppresses the proliferative capacity, migratory ability, and invasive potential of A375 human melanoma cells [23]. Using advanced techniques such as target-based screening, our research group has preliminarily demonstrated at the molecular level that alkannin acts on multiple key targets, including CPEB4 and HNRNPUL1, thereby exerting its anti-melanoma effects [24].

The CPEB family comprises a group of sequence-specific RNA-binding proteins that primarily regulate poly(A) tail length and the activation or inhibition of translation by recognizing CPE elements in the 3′-UTR of mRNAs [25,26]. CPEB4 is a key effector molecule in this family, regulating cytoplasmic polyadenylation and mRNA degradation and playing an important role in tumor-associated post-transcriptional reprogramming [27]. In melanoma, CPEB4 drives malignant tumor cell proliferation and metastasis by elevating the expression levels of critical oncoproteins, including MITF and RAB27A [28]. Since MITF is a core transcription factor in melanoma [29], and RAB family members are closely associated with intracellular transport, invasion, metastasis, and malignant progression in melanoma cells [30], CPEB4 is likely to function as a key hub molecule in the post-transcriptional regulation of the malignant phenotype of this disease. Taken together, alkannin can be considered a key candidate monomer for the antitumor effects of Arnebia euchroma. However, the in vivo pharmacodynamic profile of alkannin, the specificity of its interaction with CPEB4, and the molecular changes associated with this interaction remain to be further clarified.

## 2. Materials and Methods

### 2.1. Chemical Reagents

The chemicals and reagents used in this study are as follows: Alkannin (purity ≥ 98.0%, Cat. No. BS0783-20mg, Lot No. KB376636, Shanghai Yuanye Bio-Technology Co., Ltd., Shanghai, China), Matrigel matrix (Cat. No.: CLS356234, Corning, Inc., Corning, NY, USA), cisplatin (purity ≥ 99.0%, Cat. No.: M2223-12, Abmole Bioscience Inc., Houston, TX, USA), dimethyl sulfoxide (DMSO) (Cat. No.: 3230221001, Solarbio, Beijing, China), BCA Protein Assay Kit (Cat. No.: PC0020-50T), and SDS-PAGE Gel Preparation Kit (Cat. No.: P1200-50) were purchased from Beijing Solarbio Science Technology Co., Ltd. (Beijing, China). The CM5 sensor chip (Cat. No.: BR100530) and amine coupling kit (Cat. No.: BR100050) were purchased from Cytiva (Marlborough, MA, USA). Annexin V/7AAD (Cat. No.:AB_2869265) was purchased from BD Biosciences (Franklin Lakes, NJ, USA); CPEB4-RNAi (8201-1), CPEB4-RNAi (8204-1), and CPEB4-RNAi (8205-1) (Cat. No.: 10067525, Shanghai JiKai Gene Co., Ltd., Shanghai, China) were also used.

The following primary antibodies were used: anti-CPEB4 (Cat. No. 25342-1-AP), anti-MITF (Cat. No. 13092-1-AP), anti-PRC1 (Cat. No. 15617-1-AP), and anti-CDK1 (Cat. No. 19532-1-AP) from Proteintech Group, Inc. (Wuhan, China); anti-cleaved caspase-3 (Cat. No. GB115733-50), anti-Ki-67 (Cat. No. GB111499-50), and anti-MMP-2 (Cat. No. GB11130-50) from Servicebio Technology Co., Ltd. (Wuhan, China). HRP-conjugated goat anti-rabbit IgG (Cat. No. bs-0295G-HRP; Servicebio Technology Co., Ltd., Wuhan, China) was used as the secondary antibody.

### 2.2. Cell Culture

Human melanoma A375 cells were kindly provided by the China Type Culture Collection (Beijing, China). The cells were grown in high-glucose DMEM with 10% fetal bovine serum and antibiotics at 37 °C in a humidified environment with 5% CO_2_. Cells in the logarithmic growth phase were used for further investigation.

### 2.3. Animal Model

Sixty male specific pathogen-free (SPF) BALB/c nude mice, aged 4 to 6 weeks and weighing approximately 22 g, were acquired from Hunan Slaike Jingda Laboratory Animal Co., Ltd. The mice were maintained under standard SPF conditions at the Animal Experimentation Center of Xinjiang Medical University, which is certified under Animal Use License No. SYXK (Xin) 2018-0003. All animal procedures were approved by the IACUC of Xinjiang Medical University (No. IACUC-JT-20250311-12, Xinjiang, China).

### 2.4. Preparation of A375 Cell Suspension

A375 cells were collected for subcutaneous inoculation when 80–90% confluent. The cells were subjected to digestion with 0.25% trypsin–EDTA, thereafter washed with PBS, enumerated, and resuspended in a PBS/Matrigel combination (1:1, *v*/*v*) at a final concentration of 1 × 10^7^ cells/100 μL.

### 2.5. Establishment of a Nude Mouse Xenograft Model and Drug Administration

A total of 60 male BALB/c nude mice were included, and 10 mice were randomly selected as the normal control group without tumor cell inoculation. The remaining 50 mice were subcutaneously injected with 100 μL of A375 cell suspension into the right forelimb axillary region to establish a melanoma xenograft model. Five days after inoculation, when the subcutaneous tumors became palpable, tumor-bearing mice were randomly assigned to five groups: the model group, which received an equivalent volume of saline; the cisplatin positive control group, which received cisplatin at 2 mg/kg once every two days; and the low-, medium-, and high-dose alkannin groups, which received alkannin at 2, 3, and 4 mg/kg once daily, respectively [31]. All treatments were administered by intraperitoneal injection for 14 consecutive days. On day 15, the mice were anesthetized with sodium pentobarbital (50 mg/kg, i.p.) and euthanized via decapitation. Tumors, hearts, livers, spleens, and kidneys were excised and weighed for the calculation of tumor inhibition rates and organ coefficients [32].

### 2.6. Hematoxylin and Eosin (H&E) Staining

For histopathological analysis, six mice were randomly selected from each group. Tumor tissues and major organs were harvested, formalin-fixed, paraffin-embedded, sectioned at 4 μm, and stained with hematoxylin and eosin. The sections were examined under a light microscope to assess tumor necrosis and organ histopathology.

### 2.7. Immunohistochemistry

Immunohistochemistry was performed on tumor slices from six mice per group. After routine pretreatment, sections were incubated overnight at 4 °C with primary antibodies against Ki-67, cleaved caspase-3, and MMP-2. Signals were generated using DAB, counterstained with hematoxylin, photographed with light microscopy, and semi-quantified with ImageJ (Version 1.54 d).

### 2.8. Western Blot Analysis

Tumor proteins were extracted using RIPA buffer with protease and phosphatase inhibitors, quantified by BCA assay, separated through electrophoresis, transferred to membranes, probed with specified antibodies, and detected using ECL. Band intensities were assessed via ImageJ (Version 1.54 d).

### 2.9. Expression and Purification of Recombinant CPEB4 Protein

The pET28a-CPEB4 plasmid was transformed into BL21(DE3) competent cells using the CaCl_2_ method. Transformants were selected on kanamycin-containing agar plates, and single colonies were picked and cultured for expansion. Recombinant CPEB4 protein expression was induced with IPTG under optimized conditions. Protein expression and solubility were assessed using SDS-PAGE, Coomassie Brilliant Blue staining, and Western blot analysis. Bacterial cells induced under the optimized conditions were harvested and lysed by sonication. The recombinant CPEB4 protein was purified using Ni-NTA agarose affinity chromatography. The eluted protein was dialyzed, concentrated by ultrafiltration, filtered through a membrane, aliquoted, and stored until required.

### 2.10. DARTS

Total proteins from A375 cells were extracted and quantified as described above, then incubated with alkannin or an equal volume of DMSO at room temperature. Samples were treated with Pronase E at 0, 10, 20, 40, or 80 μg/mL for another 30 min, with 1 × TNC buffer used for the 0 μg/mL control. Target protein changes were analyzed by Western blotting.

### 2.11. CETSA

A375 cells were subjected to digestion with 0.25% trypsin–EDTA, thereafter collected, and resuspended in pre-chilled PBS supplemented with protease and phosphatase inhibitors. Cell lysates were generated through multiple freeze–thaw cycles and subsequently treated with alkannin or DMSO for 30 min at ambient temperature. Samples were heated at 37–67 °C for 5 min, then centrifuged, and the soluble proteins were examined by Western blotting.

### 2.12. SPR

SPR was conducted using a Biacore T200 device (Cytiva, Wilmington, DE, USA). Recombinant CPEB4 was affixed to a CM5 chip by amine coupling, accompanied by a blank reference channel. Alkannin was serially diluted and deposited onto the chip surface, subsequently regenerated with 10 mM NaOH for 30 s. Binding reactions and the observed KD value were assessed with Biacore T200 Evaluation software (Version 2.0).

### 2.13. Establishment of Stable CPEB4-Knockdown A375 Cells

Three lentiviral RNAi vectors targeting human CPEB4, namely LV-CPEB4-RNAi (8201-1), LV-CPEB4-RNAi (8204-1), and LV-CPEB4-RNAi (8205-1), were used to establish stable CPEB4-knockdown A375 cells. Preliminary infection experiments were performed at different multiplicities of infection and with infection-enhancing reagents to determine optimal infection conditions. Based on infection efficiency and cell growth status, LV-CPEB4-RNAi (8201-1) was selected for subsequent experiments. A375 cells were infected with LV-CPEB4-RNAi (8201-1) under the optimized condition of MOI = 10 with Hitrans GA. The medium was replaced 8 h after infection, and stable knockdown cells were selected and maintained with puromycin. Infection efficiency was evaluated by fluorescence microscopy 72 h after infection, and CPEB4 knockdown efficiency was verified by Western blot analysis.

### 2.14. CCK-8 Cell Proliferation Assay in CPEB4-Knockdown Cells

Cells were inoculated in 96-well plates at a density of 5 × 10^3^ cells per well and incubated at 37 °C with 5% CO^2^. Cells at 70–80% confluence were subjected to alkannin treatment (1–64 μM) for durations of 24 or 48 h. Blank wells contained only medium, while control wells were supplied with fresh full medium. Following treatment, CCK-8 solution was introduced and incubated for 2 h at 37 °C, after which absorbance was assessed at 450 nm.

### 2.15. Annexin V/7-AAD Apoptosis Assay

A375 cells were trypsinized, washed, resuspended in 1 × binding buffer, and adjusted to 1 × 10^6^ cells/mL. Cell suspension (100 μL) was stained with PE-conjugated Annexin V and 7-AAD for 15 min at room temperature in the dark, followed by the addition of 400 μL 1× binding buffer. Apoptosis was analyzed by flow cytometry within 1 h.

### 2.16. Cell Cycle Analysis by DNA Content Measurement

Log-phase A375 cells were treated with alkannin (2.5, 5, or 10 μM) for 24 h, with untreated cells as controls. Cells were then harvested, washed with PBS, adjusted to 1 × 10^6^ cells/mL, and fixed overnight in pre-chilled 70% ethanol at 4 °C. After ethanol removal, cells were incubated with RNase A at 37 °C for 30 min, stained with PI at 4 °C in the dark for 30 min, and analyzed for cell cycle distribution by flow cytometry.

### 2.17. Wound-Healing Assay

Cells were scratched with a sterile 200 μL pipette tip and rinsed with PBS.Cells were treated with alkannin (0, 1, 2, or 4 μmol/L), imaged at 0, 24, and 48 h, and wound closure was quantified using ImageJ (Version 1.54 d).

### 2.18. Transwell Assay

A375 and CPEB4-RNAi A375 cells were seeded in Matrigel-coated Transwell upper chambers at 5 × 10^4^ cells/mL, with 10% FBS-containing medium in the lower chambers. After 24 h, invading cells were fixed, stained with crystal violet, imaged, and quantified using ImageJ (Version 1.54 d).

### 2.19. Statistical Analysis

Data analysis was performed using GraphPad Prism 10 and SPSS 18.0. Comparisons between two groups were performed using the unpaired Student’s *t*-test, while differences across several groups were evaluated using one-way analysis of variance (ANOVA). *p* < 0.05 was considered statistically significant.

## 3. Results

### 3.1. Alkannin Inhibits Xenograft Tumor Growth in Nude Mice

**Figure 2:**
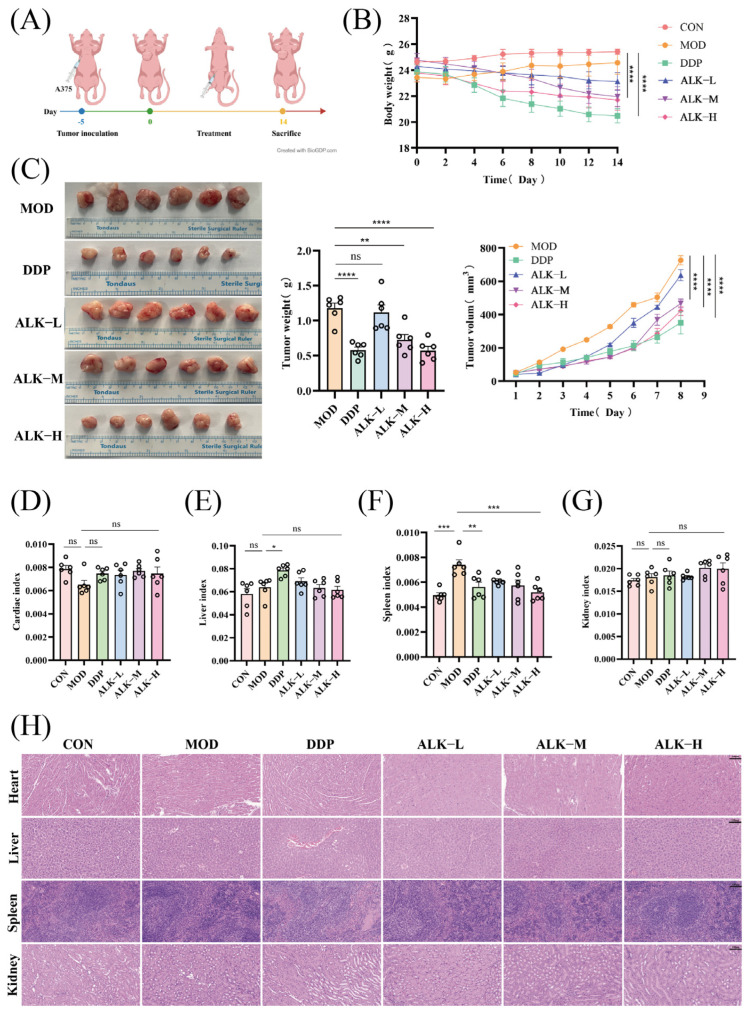
Alkannin inhibits the growth of melanoma xenografts in mice and demonstrates safety (*n* = 6): (**A**) A BALB/c mouse melanoma model was established by subcutaneous injection of 1 × 10^7^ A375 cells. (**B**) Changes in body weight in mice of each group. (**C**) Changes in tumor size, weight, and volume in each group. (**D**) Heart index. (**E**) Liver index. (**F**) Spleen index. (**G**) Kidney index. (**H**) Histomorphological changes in different tissues from mice in each group (Scale bar = 100 μm). **** *p* < 0.0001, *** *p* < 0.001, ** *p* < 0.01, * *p* < 0.05; ns indicates not significant. Data are presented as mean ± SEM.

After 14 days of administration, mice in the cisplatin and high-dose alkannin groups had a substantial decrease in body weight compared with the normal control group (*p* < 0.001), with a more pronounced reduction in the cisplatin-treated group (Figure 2B). Compared with the model group, cisplatin and medium- and high-dose alkannin markedly suppressed tumor growth, with tumor inhibition rates of 57.83%, 44.75%, and 46.36%, respectively (Table 1; Figure 2C). The spleen index was markedly elevated in the model group relative to the normal control group (*p* < 0.001; Figure 2F), while high-dose alkannin significantly diminished the spleen index compared to the model group (*p* < 0.001; Figure 2F). No discernible variations were noted in the cardiac, hepatic, or renal indices among the other groups (Figure 2D,E,G).

### 3.2. Alkannin Exhibits No Significant Organ Toxicity

H&E staining results (Figure 2H) showed that the morphological integrity of the heart and kidney tissues was preserved in all groups of mice, with no obvious pathological changes. Liver sections from the normal control group showed normal morphology, whereas hepatocyte necrosis was observed in the cisplatin group. No evident liver injury was observed in the alkannin-treated groups. Spleen sections from the normal control group and alkannin-treated groups showed largely normal morphology, whereas mild splenic sinus congestion was observed in the cisplatin group. These findings suggest that alkannin did not cause obvious histopathological alterations in the examined major organs.

### 3.3. Alkannin Inhibits Tumor Growth, Promotes Apoptosis, and Reduces Invasion-Related Marker Expression in Tumor Tissues

**Figure 3:**
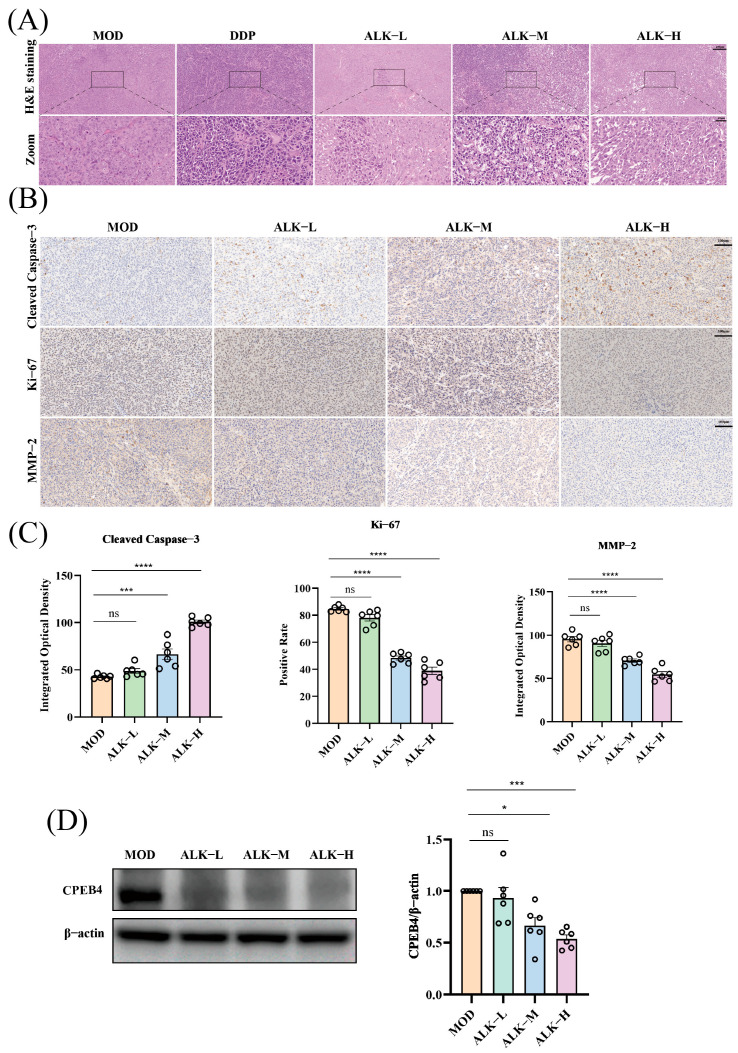
Histopathological and immunohistochemical analyses of tumor tissues (*n* = 6): (**A**) Histopathological changes in tumor tissues (Scale bar = 200 μm, 40 μm). (**B**) Immunohistochemical staining of cleaved caspase-3, Ki-67, and MMP-2 in tumor tissues (Scale bar = 100 μm). (**C**) Semi-quantitative analysis of cleaved caspase-3, Ki-67, and MMP-2. (**D**) Western blot analysis of CPEB4 protein expression in tumor tissues and semi-quantitative analysis. **** *p* < 0.0001, *** *p* < 0.001, * *p* < 0.05; ns indicates not significant. Data are presented as mean ± SEM. Original western blots can be found at Supplementary Materials.

H&E staining of tumor tissue (Figure 3A) revealed that, compared with the model group, the cisplatin group and all alkannin dose groups exhibited significant tumor cell necrosis and reduced cell density, accompanied by morphological features of cell death, including nuclear condensation and fragmentation. Immunohistochemical analysis further confirmed (Figure 3B) that the proliferation marker Ki-67 was highly expressed in the model group, whereas its expression was significantly and dose-dependently suppressed following alkannin treatment. Conversely, cleaved caspase-3, an apoptosis-related protein, was significantly upregulated in the treatment groups. Furthermore, MMP-2, an invasion-related marker, was highly expressed in tumors of the model group, whereas alkannin treatment significantly downregulated its expression in tumor tissues (*p* < 0.001; Figure 3C). Following alkannin administration, CPEB4 protein expression decreased in a dose-dependent manner. Compared with the model group, alkannin significantly downregulated relative CPEB4 protein expression in the medium-dose and high-dose groups (*p* < 0.05; Figure 3D).

### 3.4. DARTS, CETSA, and SPR Support the Interaction Between Alkannin and CPEB4

**Figure 4:**
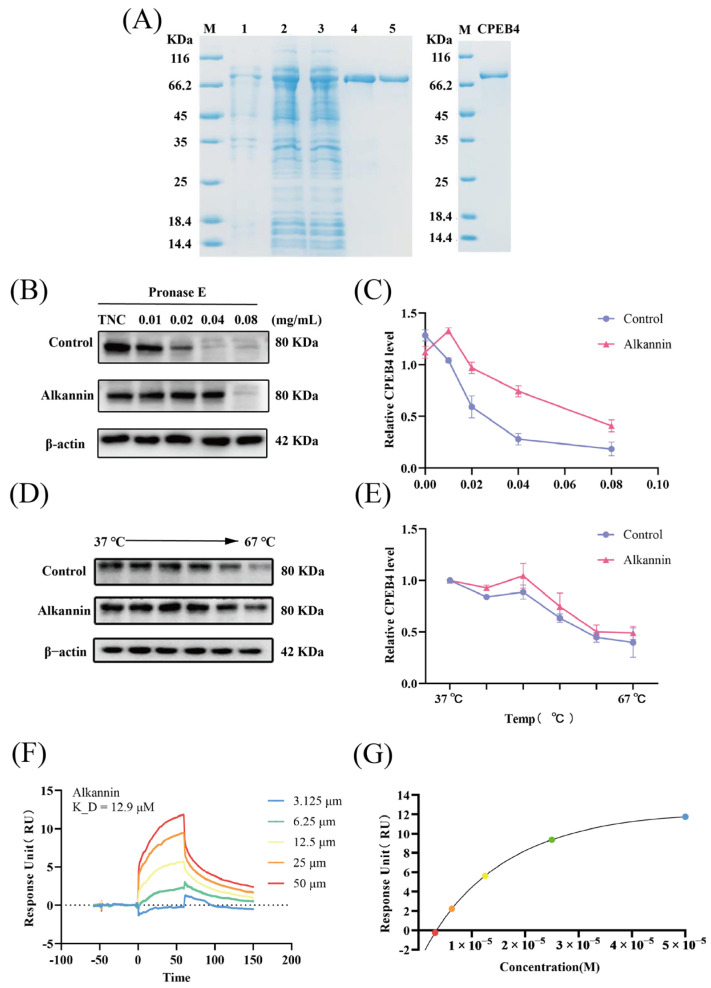
Biochemical and biophysical evidence supporting the interaction between alkannin and CPEB4: (**A**) SDS-PAGE profile of the CPEB4 protein. (**B**) DARTS assay showing the effect of alkannin on the proteolytic resistance of CPEB4. (**C**) Quantification of relative CPEB4 levels in the DARTS assay. (**D**) CETSA assay showing the effect of alkannin on the thermal stability of CPEB4. (**E**) Quantification of relative CPEB4 levels in the CETSA assay. (**F**) SPR analysis of the interaction between alkannin and CPEB4 protein. (**G**) SPR determination of the alkannin representative curve. Data are presented as mean ± SEM.

Recombinant CPEB4 protein was successfully expressed and showed a distinct target band in the eluate (Figure 4A). In the target-binding validation assays, DARTS analysis showed that Pronase E reduced CPEB4 protein abundance in a concentration-dependent manner. However, alkannin treatment significantly protected CPEB4 from enzymatic degradation at different Pronase E concentrations (*p* < 0.05; Figure 4B,C). Similarly, CETSA analysis showed that CPEB4 protein gradually decreased as temperature increased from 37 to 67 °C, whereas alkannin treatment significantly attenuated the thermal denaturation of CPEB4 and enhanced its thermal stability (*p* < 0.05; Figure 4D,E). SPR analysis further demonstrated concentration-dependent physical binding of alkannin to CPEB4 over a concentration range of 3.125–50 μM, with a micromolar binding affinity (K_D = 12.9 μM; Figure 4F,G).

### 3.5. CPEB4 Knockdown Increases the Sensitivity of A375 Cells to the Antiproliferative, ProApoptotic, and Cell Cycle Effects of Alkannin

**Figure 5:**
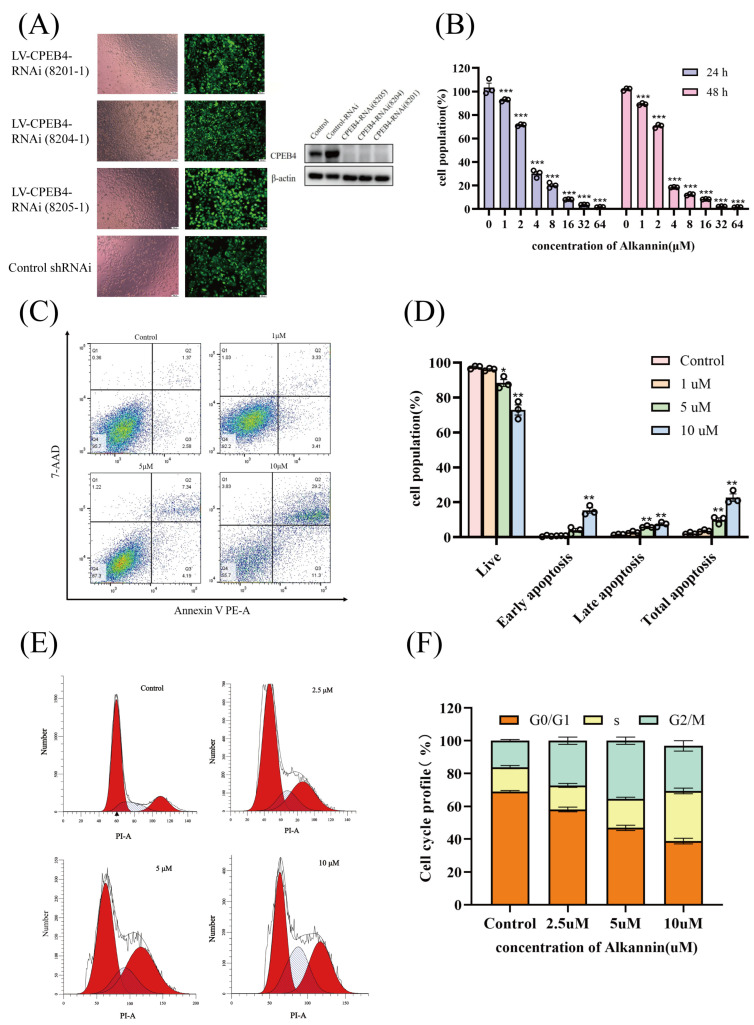
Effects of alkannin on proliferation, apoptosis, and cell cycle progression in A375 cells after CPEB4 knockdown: (**A**) Transfection efficiency was assessed in melanoma A375 cells, and Western blot analysis was performed to determine the endogenous CPEB4 protein expression level after RNA interference (Scale bar = 100 μm). (**B**) Effects of alkannin on the proliferative activity of A375 cells after CPEB4 knockdown at different time points (*n* = 5). (**C**) Cell apoptosis was analyzed by Annexin V/7-AAD double staining (*n* = 3). (**D**) Percentages of viable and apoptotic cells. (**E**) Cells were treated with alkannin for 24 h, stained with propidium iodide, and analyzed by flow cytometry (*n* = 3). (**F**) Proportions of cells in the G0/G1, S, and G2/M phases of the cell cycle (*n* = 3). *** *p* < 0.001, ** *p* < 0.01, * *p* < 0.05; ns indicates not significant. Data are presented as mean ± SEM.

A375 cells were successfully infected with three lentiviral CPEB4-RNAi vectors, including CPEB4-RNAi (8201-1), CPEB4-RNAi (8204-1), and CPEB4-RNAi (8205-1), all of which showed high infection efficiency. Among them, CPEB4-RNAi (8201-1) exhibited high infection efficiency and good cell growth status and was therefore selected for subsequent experiments (Figure 5A). Western blot analysis showed that CPEB4-RNAi (8201-1), CPEB4-RNAi (8204-1), and CPEB4-RNAi (8205-1) markedly reduced endogenous CPEB4 protein expression compared with the control and negative control shRNA groups (Figure 5B).

The CCK-8 assay demonstrated that CPEB4 knockdown markedly enhanced A375 cell sensitivity to alkannin. In the CPEB4-knockdown group, the 24 h IC_50_ value was 2.83 ± 0.07 μM, and treatment with 8 μM alkannin resulted in an inhibition rate close to 90% (*p* < 0.05; Figure 5B). Annexin V/7-AAD staining showed that 10 μM alkannin induced marked apoptosis in A375 cells, with a total apoptosis rate of 40.84% and an early apoptosis rate of 29.2%. This apoptotic effect was more pronounced in CPEB4-knockdown cells than in non-knockdown control cells, suggesting that CPEB4 downregulation may enhance alkannin-induced apoptosis in A375 cells (*p* < 0.05; Figure 5C,D). Cell cycle analysis showed that alkannin treatment induced S-phase and G2/M-phase arrest in CPEB4-knockdown cells, with a stronger effect than in control cells (*p* < 0.05; Figure 5E,F).

### 3.6. CPEB4 Knockdown Enhances the Inhibitory Effects of Alkannin on A375 Cell Migration and Invasion

**Figure 6:**
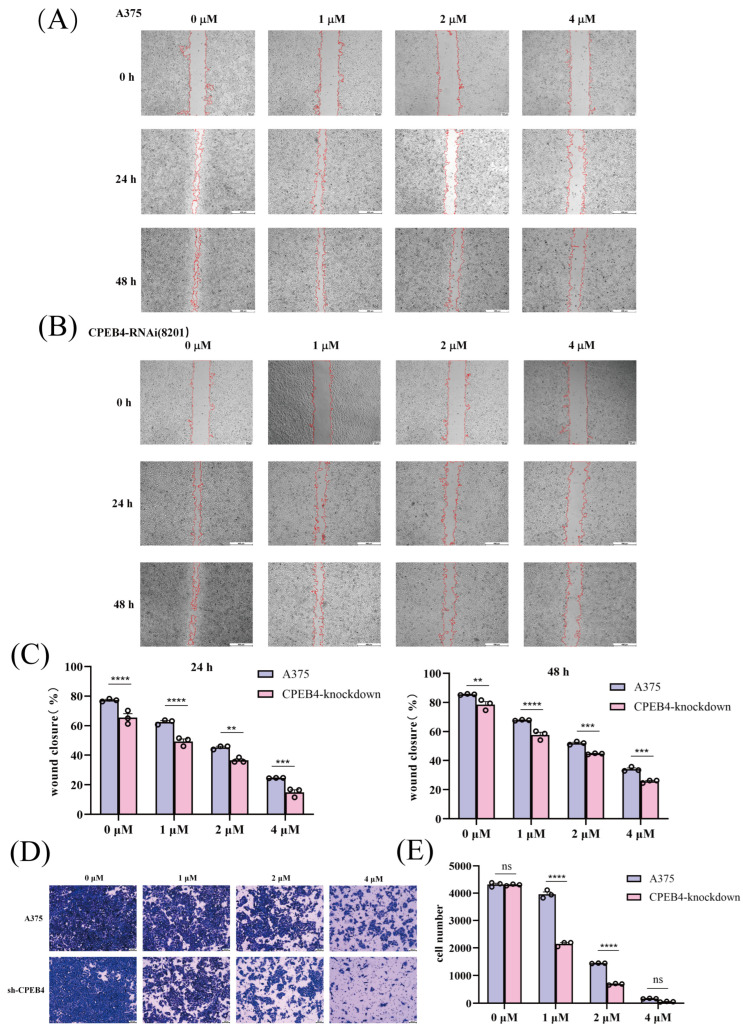
CPEB4 knockdown enhances the inhibitory effects of alkannin on the migration and invasion of A375 cells in vitro. (*n* = 3): (**A**) Wound-healing assay showing the effect of alkannin on the migratory capacity of A375 cells (o h scale bar = 100 μm; 24 and 48 h scale bar = 498 μm). (**B**) Wound-healing assay showing the effect of alkannin on the migratory capacity of A375 cells after CPEB4 knockdown (o h scale bar = 100 μm; 24 and 48 h scale bar = 498 μm). (**C**) Quantitative analysis of wound closure rates in A375 cells in the control group and the CPEB4 knockdown group at different time points. (**D**) Transwell invasion assay showing the effect of alkannin on the invasive capacity of A375 cells before and after CPEB4 knockdown (Scale bar = 100 μm). (**E**) Quantitative analysis of the number of invaded cells in each group. **** *p* < 0.0001, *** *p* < 0.001, ** *p* < 0.01; ns indicates not significant. Data are presented as mean ± SEM.

The wound-healing assay showed that alkannin inhibited A375 cell migration in a dose-dependent manner at 0, 1, 2, and 4 μM. This inhibitory effect was more pronounced in CPEB4-knockdown cells than in control A375 cells (Figure 6A,B). After treatment with 4 μM alkannin for 24 h, the wound closure rate was 25% in control A375 cells and 15% in CPEB4-knockdown cells. This trend persisted after 48 h, indicating that CPEB4 downregulation enhanced the inhibitory effect of alkannin on cell migration (*p* < 0.05; Figure 6C).

Transwell assay results further showed that alkannin suppressed A375 cell invasion. At 4 μM alkannin, the average number of invading cells decreased to 170 in control A375 cells and further decreased to 57 in CPEB4-knockdown cells. At the same alkannin concentration, CPEB4-knockdown cells consistently showed fewer invading cells than control A375 cells (*p* < 0.05; Figure 6D,E).

### 3.7. Changes in CDK1, PRC1, and MITF Expression Following Alkannin Treatment and CPEB4 Knockdown

**Figure 7:**
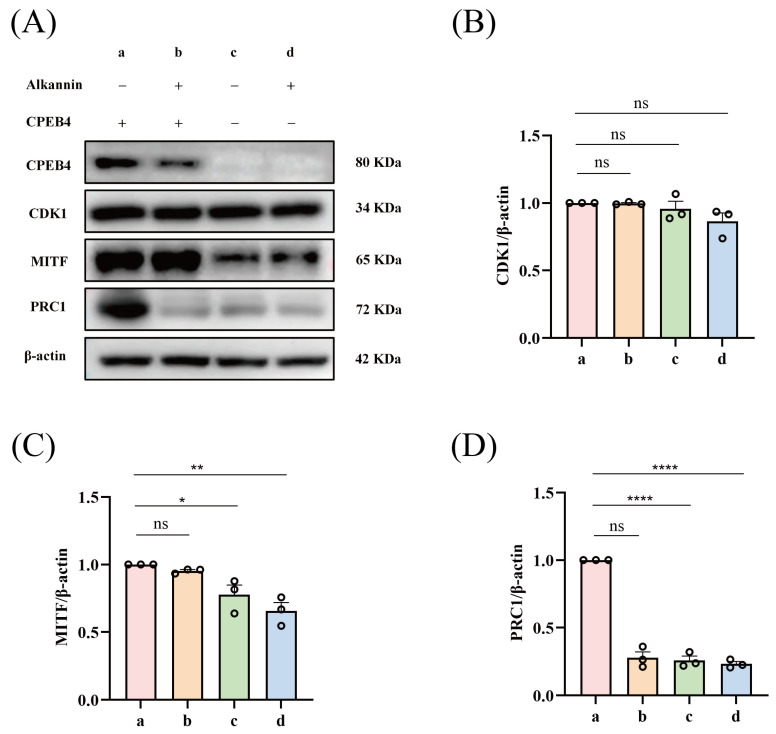
Expression levels of CDK1, MITF, and PRC1 proteins after alkannin treatment and CPEB4 knockdown (*n* = 3): (**A**) Western blot analysis of protein bands. (**B**) Effects of alkannin on CDK1 protein expression. (**C**) Effects of alkannin on MITF protein expression. (**D**) Effects of alkannin on PRC1 protein expression. The horizontal axes: (**a**) untreated, CPEB4-positive group. (**b**) Alkannin-treated, CPEB4-positive group. (**c**) untreated, CPEB4-negative group. (**d**) Alkannin-treated, CPEB4-negative group. **** *p* < 0.0001, ** *p* < 0.01, * *p* < 0.05; ns indicates not significant. Data are presented as mean ± SEM.

Western blot analysis showed that alkannin treatment and CPEB4 knockdown both significantly reduced CPEB4 protein expression in A375 cells compared with untreated normal A375 cells (*p* < 0.05; Figure 7A). Alkannin treatment alone and CPEB4 knockdown alone had no significant effect on CDK1 expression (*p* > 0.05; Figure 7A,B) For MITF, alkannin treatment alone did not significantly alter its expression, while MITF expression was significantly reduced in both CPEB4-knockdown cells and alkannin-treated CPEB4-knockdown cells (*p* < 0.05; Figure 7A,C). PRC1 expression was significantly decreased after alkannin treatment, CPEB4 knockdown, and alkannin treatment combined with CPEB4 knockdown (*p* < 0.05; Figure 7A,D).

## 4. Discussion

In this study, a nude mouse subcutaneous xenograft model was established to evaluate the in vivo antitumor activity of alkannin against melanoma. Alkannin inhibited xenograft tumor growth, with the medium- and high-dose groups exhibiting more pronounced tumor-suppressive effects. Histopathological examination of major organs revealed no obvious pathological injury in the alkannin-treated groups, suggesting that alkannin exerted antitumor activity without causing apparent histopathological damage in the examined organs under the present experimental conditions.

H&E staining and immunohistochemical analysis of tumor tissues further supported the antitumor effect of alkannin. Tumor tissues from alkannin-treated mice exhibited obvious tumor cell necrosis and reduced cell density. Moreover, alkannin administration reduced Ki-67 levels, a marker of cell proliferation, while enhancing cleaved caspase-3 expression, a marker of apoptosis. The findings indicate that the in vivo anticancer efficacy of alkannin is partially mediated by suppression of tumor cell growth and enhancement of apoptosis. Similar antitumor effects have also been reported for shikonin-related naphthoquinones in melanoma models [33]. The present study also showed that alkannin reduced MMP-2 expression in tumor tissues. MMP-2 is involved in extracellular matrix degradation and is closely associated with tumor invasion, metastasis, and remodeling of the tumor microenvironment [34,35]. Therefore, decreased MMP-2 expression suggests that alkannin may suppress invasion-related phenotypes in melanoma. However, because the in vivo model used in this study was a subcutaneous xenograft model rather than a metastasis model, the anti-invasive and anti-metastatic effects of alkannin require further validation using appropriate metastatic melanoma models.

Previous target-fishing studies identified CPEB4 as a potential alkannin-binding protein in A375 cells [24]. Based on this finding, the present study further employed DARTS, CETSA, and SPR assays to validate the interaction between alkannin and CPEB4. DARTS showed that alkannin protected CPEB4 from enzymatic degradation; CETSA demonstrated that alkannin enhanced the thermal stability of CPEB4; and SPR confirmed direct binding between alkannin and CPEB4 with micromolar affinity. Together, these results support CPEB4 as a candidate direct-binding target of alkannin. Although DARTS and CETSA showed ligand-induced stabilization of CPEB4, this does not necessarily conflict with the reduced steady-state CPEB4 protein level observed after sustained alkannin treatment. CPEB4 reduction was observed in both xenograft tumors and alkannin-treated A375 cells; it cannot be attributed solely to reduced tumor burden and may instead involve altered protein homeostasis, post-transcriptional regulation, and changes in cellular proliferation.

CPEB4 has been shown to perform a lineage-specific function in melanoma and to modulate melanoma-associated pathways, including MITF and RAB27A [28]. The current study demonstrated that CPEB4 knockdown amplified the inhibitory effects of alkannin on A375 cell proliferation, migration, and invasion, while also facilitating alkannin-induced apoptosis and cell cycle arrest. These findings indicate that CPEB4 status influences melanoma-associated phenotypes and modulates cellular sensitivity to alkannin. Notably, the enhanced response observed after CPEB4 knockdown does not establish that alkannin activity is functionally dependent on CPEB4. Rather, the results suggest that reduced CPEB4 expression may alter the malignant cellular state, thereby increasing susceptibility to alkannin treatment. Further rescue experiments will be required to establish target-specific functional causality. MITF is a key melanoma-associated transcription factor involved in melanoma lineage maintenance, proliferation, and phenotypic plasticity. In this study, alkannin treatment alone did not significantly alter MITF expression, whereas CPEB4 knockdown significantly reduced MITF protein expression. These findings suggest an association between MITF expression and CPEB4 status in A375 cells but do not support a simple linear alkannin–CPEB4–MITF regulatory axis under the present experimental conditions. The different effects observed after alkannin treatment and stable CPEB4 knockdown may reflect differences in the magnitude or duration of CPEB4 modulation. Therefore, although CPEB4 may regulate MITF expression, whether MITF contributes to the CPEB4-associated antitumor effects of alkannin remains to be mechanistically validated. In addition to MITF, this study examined the expression of cell cycle-related proteins, including CDK1 and PRC1. PRC1 is a microtubule-associated protein involved in spindle midzone organization and cytokinesis [36,37]. In melanoma, PRC1 has also been implicated in cell cycle regulation and differentiation through a noncoding RNA/miRNA-dependent mechanism [38]. In the present study, PRC1 was consistently reduced by alkannin treatment, CPEB4 knockdown, and their combination, making it the most concordant molecular readout among the proteins examined. Given that CPEB4 is an RNA-binding protein involved in post-transcriptional regulation and mitotic mRNA localization and translation, these parallel changes raise the possibility that PRC1 may participate in a CPEB4-associated post-transcriptional regulatory network. CDK1 is an important regulator of cell cycle progression, particularly the G2/M transition [39]. In this study, neither alkannin treatment alone nor CPEB4 knockdown alone significantly altered CDK1 expression. Thus, the present data do not establish CDK1 as a primary downstream mediator of alkannin-induced G2/M arrest. Because CDK1 activity is strongly influenced by its phosphorylation status and association with cyclin partners, further analyses of phospho-CDK1, cyclin B1, and other G2/M regulatory proteins will be required to clarify this mechanism [40]. Previous studies have reported that alkannin can induce G2/M-phase arrest through mechanisms involving GSK3β-related signaling, suggesting that alkannin-induced cell cycle arrest may involve multiple regulatory pathways [31].

Although alkannin directly binds to CPEB4, CPEB4 is a functional regulatory node that supports melanoma-associated oncogenic programs. Therefore, genetic knockdown of CPEB4 may weaken the proliferative, migratory, invasion-related, and mitotic regulatory capacities of A375 cells, thereby rendering them more vulnerable to additional pharmacological stress induced by alkannin. In this context, alkannin may further perturb residual CPEB4-dependent signaling and CPEB4-associated downstream regulatory networks, thereby enhancing the cellular sensitivity of melanoma cells to its therapeutic effects. Thus, the available findings support an interaction between alkannin and CPEB4 and suggest that CPEB4-associated regulatory changes may contribute to the cellular response to alkannin, rather than indicating a simple one-target/one-effect mechanism.

## 5. Conclusions

In conclusion, alkannin demonstrated antitumor activity against melanoma in vivo and a favorable preliminary safety profile, as assessed by histopathological examination of major organs. Mechanistically, alkannin directly binds to CPEB4 and may suppress melanoma cell proliferation, migration, and invasion by interfering with CPEB4-associated post-transcriptional regulatory processes. Stable CPEB4 knockdown was associated with reduced MITF expression, whereas PRC1 was consistently downregulated following alkannin treatment, CPEB4 knockdown, and their combination, suggesting that distinct CPEB4-associated regulatory processes may contribute to the antitumor effects of alkannin. These findings support CPEB4 as an important molecular target involved in the anti-melanoma activity of alkannin and provide experimental evidence for further mechanistic studies and potential drug development. Nevertheless, in vivo validation using CPEB4-knockdown or CPEB4-intervention models remains lacking, and the precise relationships among alkannin, CPEB4, MITF, and PRC1 require further investigation.

## Figures and Tables

**Table 1:** Comparison of tumor inhibition rates of alkannin in melanoma-bearing mice (*** *p* < 0.001, x ± s, *n* = 6).

Group	Tumor Weight (g)	Tumor Inhibition Rate
Model	0.817 ± 0.526	-
DDP	0.345 ± 0.211 ***	57.83%
ALK-L	0.625 ± 0.518	23.43%
ALK-M	0.451 ± 0.366 ***	44.75%
ALK-H	0.438 ± 0.403 ***	46.36%

## Data Availability

The data that support the findings of this study are available from the corresponding author upon reasonable request.

## Supplementary Materials

The following supporting information can be downloaded at: https://www.mdpi.com/article/10.3390/biom16071064/s1, Figure S1: Original Western Blots Figure 3D CPEB4 and β-Actin; Figure S2: Original Western Blots Figure 4B Control, Alkannin and β-Actin; Figure S3: Original Western Blots Figure 4D Control, Alkannin and β-Actin; Figure S4: Original Western Blots Figure 5B CPEB4 and β-Actin; Figure S5: Original Western Blots Figure 7A CPEB4, CDK1, MITF, PRC1 and β-Actin.

## Abbreviations

The following abbreviations are used in this manuscript:

i.p.	Intraperitoneal
MOD	Model
CON	Control
DDP	Cisplatin
ALK-L	Alkannin—low-dose
ALK-M	Alkannin—medium-dose
ALK-H	Alkannin—high-dose
