# Supplementary material for: Interaction of Alkannin with CPEB4 Contributes to Its Antitumor Effects in Melanoma
Source: Biomolecules. 2026 Jul 21;16(7):1064. doi: 10.3390/biom16071064 (PMC13406732; doi:10.3390/biom16071064)

Figure S1: Figure 3 D
CPEB4

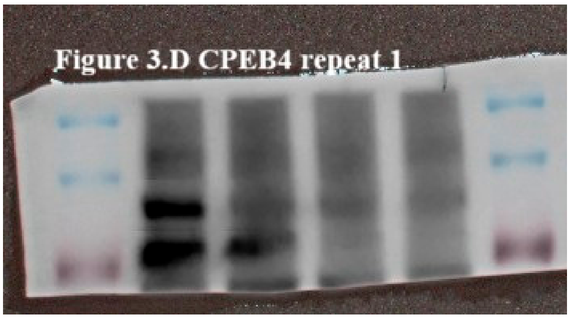

β-Actin

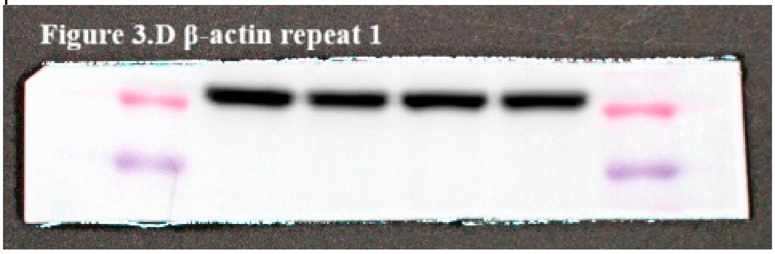

Figure S2: Figure 4 B
Control; Alkannin

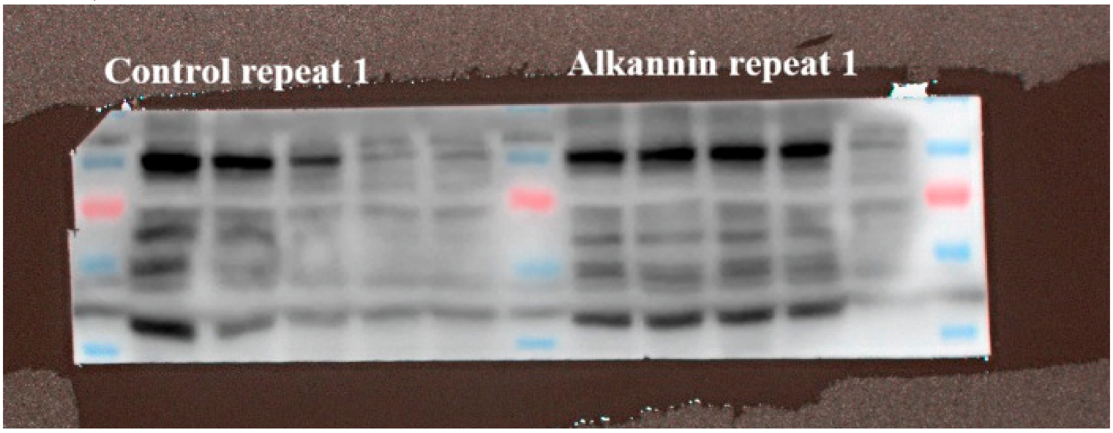

β-Actin

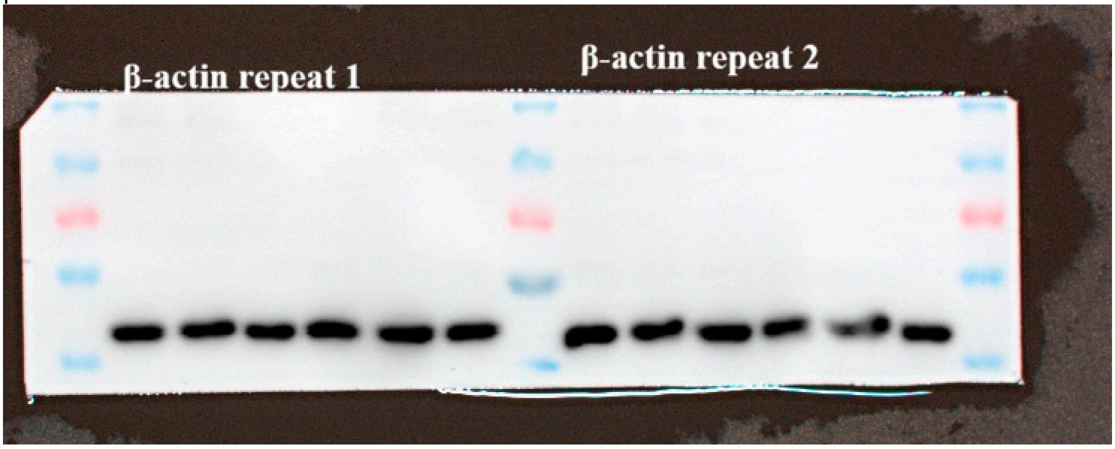

Figure S3: Figure 4 D
Control; Alkannin

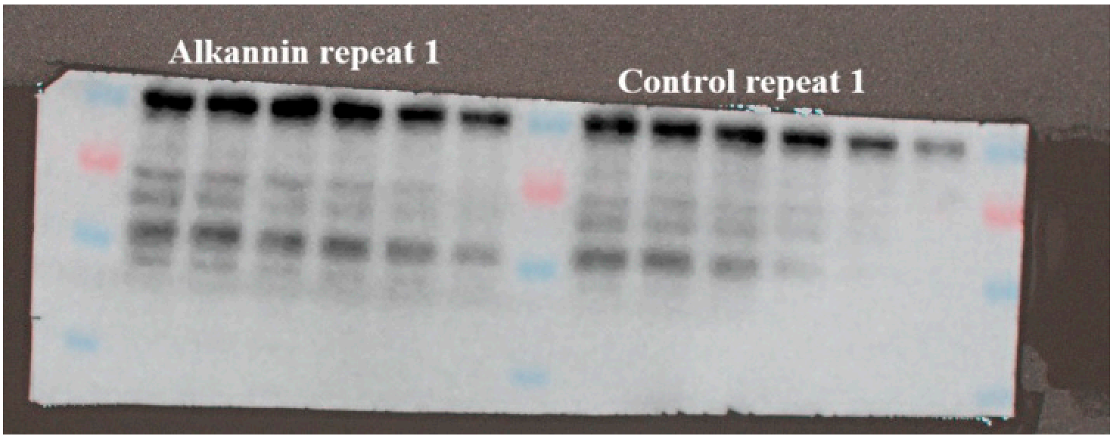

β-Actin

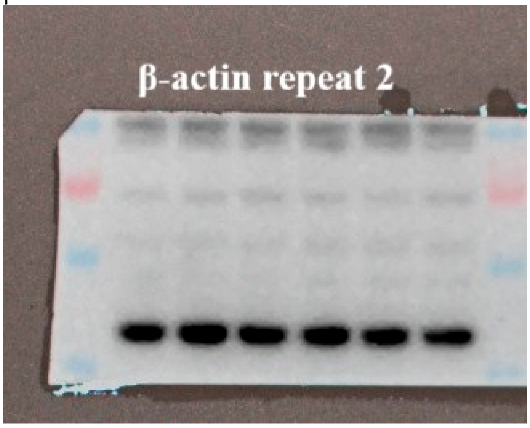

Figure S4: Figure 5 B

CPEB4

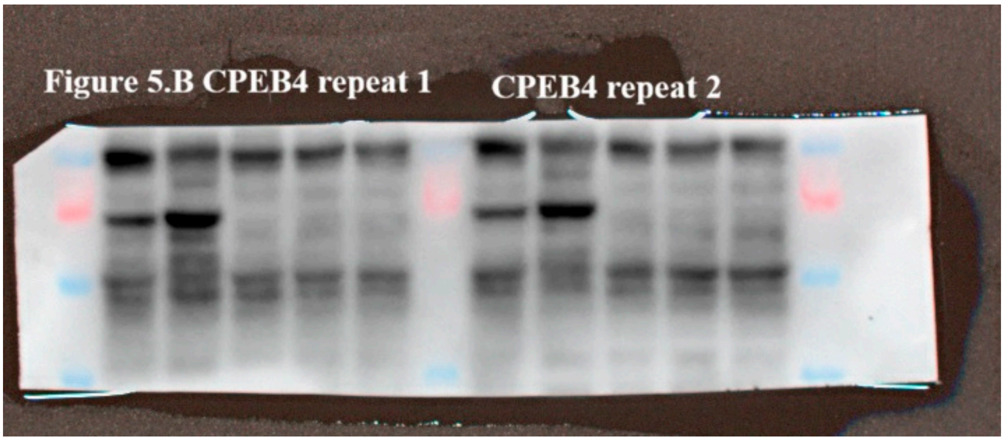

β-actin

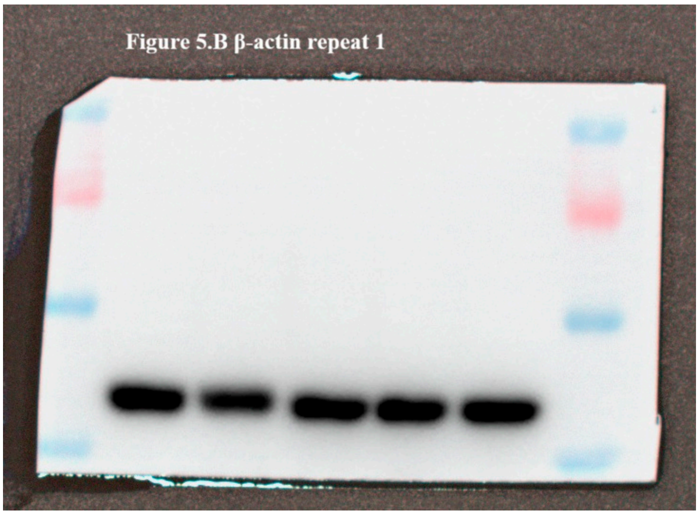

Figure S5: Figure 7 A

CPEB4

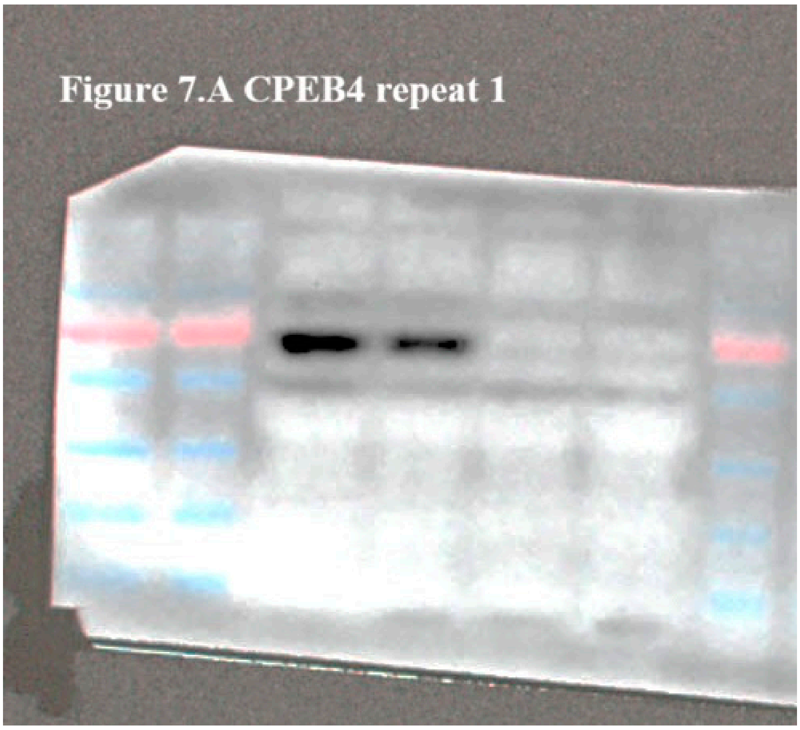

CDK1

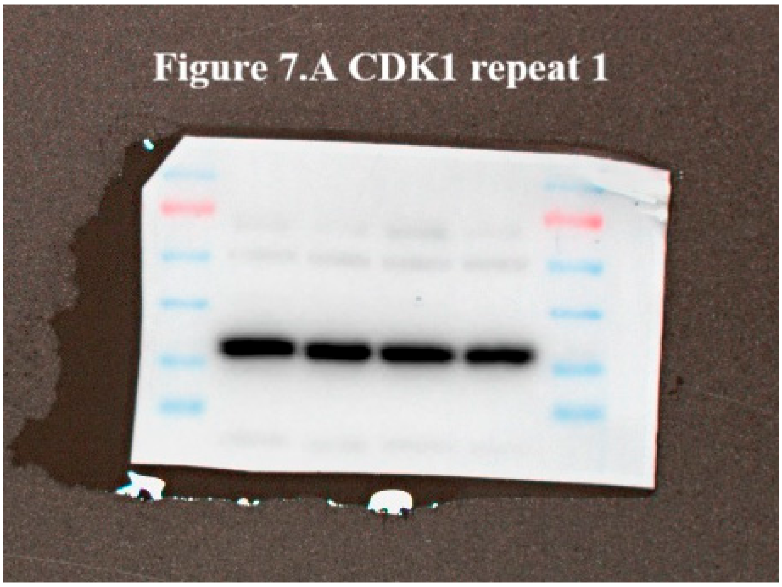

MITF

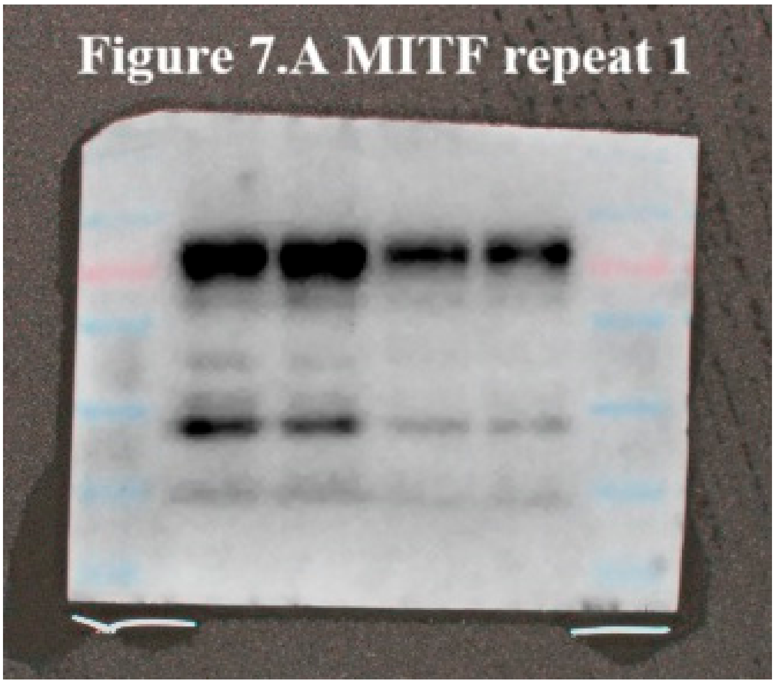

PRC1

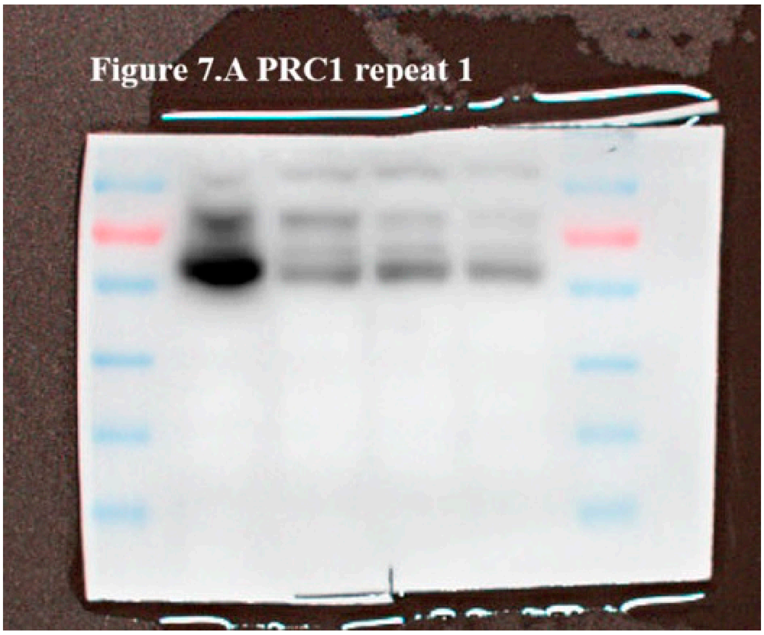

β-Actin

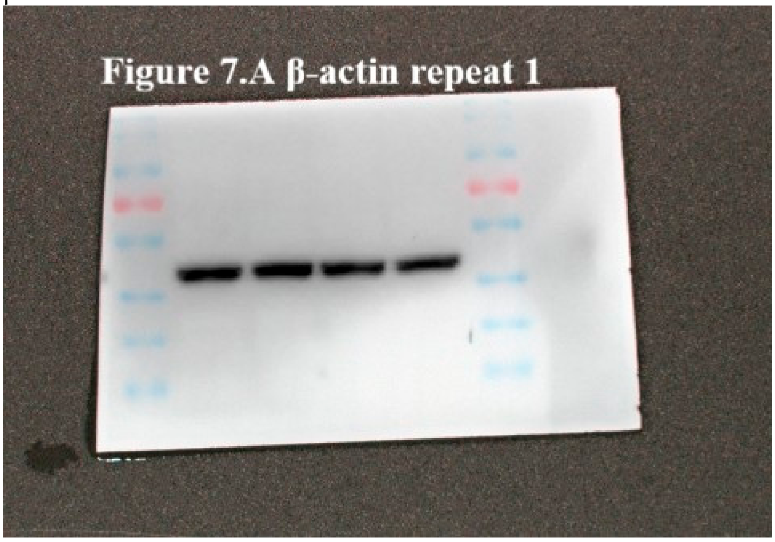

Supplement: Supplementary file 1 [file biomolecules-16-01064-s001.zip › biomolecules-4392322-supplementary.pdf]
